# The role of MDCT in the diagnosis of primary pericardial tumours: a case report and review of literature

**DOI:** 10.1259/bjrcr.20150028

**Published:** 2015-06-10

**Authors:** A Aggarwal, A K Gupta, A Kapoor Aggarwal

**Affiliations:** Department of Radiodiagnosis, Shri Ram Murti Smarak Institute of Medical Sciences, Bareilly, Uttar Pradesh, India

## Abstract

Primary pericardial tumours are very rare and are hence not usually part of our differential diagnosis, especially since they have non-specific signs and symptoms. While chest radiography remains the most common initial imaging investigation in the assessment of suspected cardiothoracic pathology, the diagnostic yield for assessing pericardial lesions is limited, often necessitating the need for further assessment with echocardiography, CT scan or MRI. We present a case of an adult male patient with an incidental primary pericardial tumour diagnosed during the routine imaging assessment of suspected pulmonary infections. After proper formulation of diagnosis, the patient was managed accordingly for pulmonary pathology and discharged on recovery. Over the years, with advancement and widespread increase in use of multidetector CT and MRI, diagnosing primary pericardial tumours has become easier. MRI has now become the modality of choice for imaging of pericardial tumours because of its better soft-tissue contrast resolution.

## Summary

Prevalence rate of primary pericardial tumours is low, approximately 0.02–0.056% with the tumours arising primarily from pericardium even rarer.^1,2^ Patients with primary pericardial neoplasms present with diverse signs and symptoms that are usually the result of associated pericardial effusion, pericarditis or invasion of adjacent structures. Primary pericardial tumour may be found incidentally during work-up for an unrelated illness in asymptomatic patients.^2,3^ We present a case where the patient presented with similar non-specific symptoms, which were found most likely not to be directly related to the presence of primary pericardial tumour. Although the finding of a primary pericardial tumour was incidental to our patient’s clinical presentation, it resulted in a diagnostic dilemma, which in the absence of cross-sectional imaging assessment might have been misdiagnosed.

## Clinical presentation

A 61-year-old male patient presented to the Chest & Pulmonary Medicine outpatient clinic of our hospital with complaints of breathlessness, cough and fever for a fortnight. Fever was low grade, continuous and decreased on taking medication, whereas cough was predominantly dry in nature. Cardiovascular, central nervous systems and abdominal examinations were within normal limits. Based on these clinical findings, a provisional diagnosis of an acute pulmonary infection was made. Routine haematological and serological investigations were performed along with a chest radiograph.

## Imaging findings

**Figure 1.**
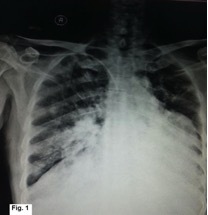
Chest radiograph showing cardiomegaly and bilateral consolidation.

**Figure 2.**
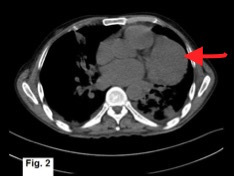
Axial non-contrast CT image showing a large left pericardial lesion.

**Figure 3.**
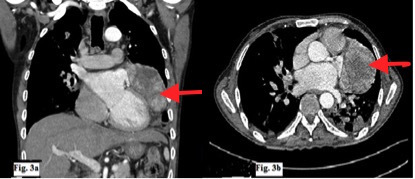
(a,b) Coronal reformatted and axial contrast-enhanced CT image showing left-sided pericardial lesion with heterogeneous enhancement. Non-enhancing hypodense areas are seen within the lesion suggestive of necrotic areas. No obvious invasion of underlying cardiac structures is seen.

The chest radiograph demonstrated multifocal bilateral consolidation, along with cardiomegaly (Figure 1). Echocardiography, however, was unremarkable with no demonstrable pericardial abnormality. Contrast-enhanced CT confirmed the presence of bilateral consolidation but also demonstrated a large broad-based soft-tissue mass along the left heart border extending superiorly up to the left hila (Figures 2, 3a). The fat planes of the mass lesion with the adjacent myocardium, pleura and lung parenchyma were well maintained with no evidence of invasion of any adjacent structure (Figure 3b). A delayed (50 s after injection of contrast) contrast scan showed heterogeneous enhancement of the lesion. Cardiac structures otherwise showed normal contrast enhancement. Cardiac MRI was not performed as it is not available at our institute.

## Treatment

**Figure 4.**
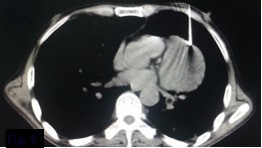
Soft-tissue window image of CT-guided biopsy.

**Figure 5.**
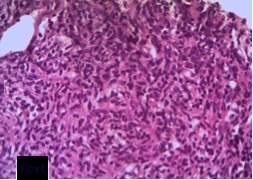
Histopathological slide showing multiple spindle-shaped cells.

CT-guided fine needle aspiration cytology and biopsy (Figure 4) of the lesion revealed proliferation of fibrous tissue in a sheet-like pattern, which were elongated, spindle shaped with elongated bland nuclei (Figure 5). No mitotic activity was seen. Based on these findings, a diagnosis of primary benign mesenchymal tumour of pericardium was made, with fibroma and benign mesothelioma being the primary differentials. 

## Management and outcome

Because of the severity of pneumonia and lack of response to the first line of antibiotic therapy, the patient required a short period of clinical stabilization on the intensive care unit. After successful treatment of the patient’s pneumonia, the patient was discharged. Because of comorbidity, the patient was deemed unsuitable for surgical resection of the pericardial fibroma, particularly given the fact that the patient remained asymptomatic at discharge. The patient will remain under regular clinical follow-up.

## Differential diagnosis

Fibromas are rare primary pericardial mesenchymal tumours. Characteristically, they are benign tumours.^4^ Studies have shown that fibromas show heterogeneous contrast enhancement on post-contrast images, as also seen in our case.^5^ Morphologically, in our case, pericardial paraganglioma was a close differential diagnosis, especially the non-functioning type. Pericardial paragangliomas are also usually benign lesions and non-functioning, with functioning counterparts being very rare.^2,6^ On chest radiographs, they also appear as left-sided cardiac enlargement or as a mass splaying the carina. Contrast-enhanced CT examination usually reveals hyperdense enhancement of these lesions with central necrotic areas. However, easier imaging diagnosis with use of nuclear imaging has been shown, with increased uptake shown by paragangliomas on examination with indium-111-pentetreotide or I-131 metaiodobenzylguanidine scan (MIBG) or I-123 MIBG.^2,6^

## Discussion

Primary pericardial lesions are classified as benign or malignant. The most common benign lesions are pericardial cysts and lipomas, whereas mesothelioma is the most common malignant primary pericardial tumour.^5^ Primary pericardial tumours present with non-specific signs and symptoms, with dyspnoea, chest pain, palpitations, fever and weight loss being the most common presenting symptoms. The patient’s clinical history is also very valuable in narrowing of differential diagnosis, for example, in a patient with known malignant neoplasm, pericardial lesion will most likely be a metastatic tumour. 

Although chest radiographs and echocardiography are the most commonly utilized imaging tools to evaluate patients with suspected pericardial pathology, cross-sectional imaging is often required to diagnose pericardial tumours. While cardiac MRI is the cross-sectional imaging technique of choice in evaluating for suspected pericardial tumours, multidetector CT has also been shown to be reliable in the assessment of these patients and is particularly useful in institutions where cardiac MRI might not be as readily accessible.^7–9^ Positron emission tomography/CT scan may be used as a staging tool in advanced malignancies.^2^ While recognizing the key imaging appearances of primary pericardial tumours is important, histological evaluation is almost always required to confirm the diagnosis and guide further management.^5^

CT scan and MRI both provide detailed assessment of the site of the pericardial tumour, tissue characteristics, extent of local invasion and relationship of the lesion to underlying structures that may contraindicate curative surgery.^7–9^ Both CT scan and MRI may differentiate benign from aggressive lesions and pericardial lipomas and cysts from neoplasms.^7–9^ Cardiac MRI provides better contrast resolution and more accurate assessment of myocardial invasion in comparison to CT scan.^2^ This, along with the avoidance of ionizing radiation exposure, makes cardiac MRI the cross-sectional imaging modality of choice when evaluating suspected pericardial lesions.^7–9^

Along with its usefulness in making a diagnosis, imaging also plays an important role in assessing the resectability of these lesions and evaluating the patient for associated complications. Some of the commonly seen complications include invasion of mediastinal structures, regional or distant metastases, pericardial effusion, cardiac tamponade, compression of vascular structures or cardiac chambers, encasement of vital structures, involvement of coronary arteries, myocardial infarction, diastolic dysfunction and constrictive physiology.^2^ Pericardial fibromas have also been mentioned as presenting with palpitations, possibly because of their large size during presentation.^4,5^

## Learning points

1. Primary pericardial neoplasms are rare but are relevant tumours.
2. Benign pericardial lesions may cause diagnostic dilemmas that are potentially resolvable by the appropriate use of cross-sectional imaging.
3. In cases of malignant lesions, CT scan and MRI are of utmost importance in assessing the extent of invasion of these tumours and the extent of compression of cardiac chambers, pericardial effusion, constrictive physiology and locoregional or distant metastatic disease.

## References

[1] Lam KY, Dickens P, Chan AC. Tumors of the heart. A 20-year experience with a review of 12,485 consecutive autopsies. Arch Pathol Lab Med 1993; 117: 1027–31. 8215825

[2] Restrepo CS, Vargas D, Ocazionez D, Martínez-Jiménez S, Betancourt Cuellar SL, Gutierrez FR. Primary pericardial tumors. Radiographics 2013; 33: 1613–30. 24108554 10.1148/rg.336135512

[3] Lamba G, Frishman WH. Cardiac and pericardial tumors. Cardiol Rev 2012; 20: 237–52. 22447042 10.1097/CRD.0b013e31825603e7

[4] Randhawa K, Ganeshan A, Hoey ET. Magnetic resonance imaging of cardiac tumors: part 1, sequences, protocols, and benign tumors. Curr Probl Diagn Radiol 2011; 40: 158–68. 21616278 10.1067/j.cpradiol.2010.07.001

[5] Efthymiou C, Abu-Omar Y, Ratnatunga C. Massive intrapericardial fibroma Jpresenting with palpitations. Heart 2007; 93: 106. 17170346 10.1136/hrt.2006.087965 PMC1861360

[6] Intezo CM, Jabbour S, Lin HC, Miller JL, Kim SM, Capuzzi DM, et al. Scintigraphic imaging of body neuroendocrine tumors. Radiographics 2007; 27: 1335–69. 17848696 10.1148/rg.275065729

[7] O’Leary SM Williams PL Williams MP Edwards AJ Roobottom CA Morgan-Hughes GJ et al Imaging the pericardium: appearances on ECG-gated 64-detector row cardiac computed tomography. Br J Radiol 2010; 83: 194–205. 20197434 10.1259/bjr/55699491 PMC3473560

[8] Peebles CR, Shambrook JS, Harden SP. Pericardial disease—anatomy and function. Br J Radiol 2011; 84: S324–S337. 22723538 10.1259/bjr/16168253 PMC3473919

[9] Rajiah P, Kanne JP. Computed tomography of the pericardium and pericardial Jdisease. J Cardiovasc Comput Tomogr 2010; 4: 3–18. 20159622 10.1016/j.jcct.2010.01.004

